# PreImplantation factor (PIF) protects cultured embryos against oxidative stress: relevance for recurrent pregnancy loss (RPL) therapy

**DOI:** 10.18632/oncotarget.16028

**Published:** 2017-03-08

**Authors:** Lindsay F. Goodale, Soren Hayrabedran, Krassimira Todorova, Roumen Roussev, Sivakumar Ramu, Christopher Stamatkin, Carolyn B. Coulam, Eytan R. Barnea, Robert O. Gilbert

**Affiliations:** ^1^ Department of Clinical Sciences, College of Veterinary Medicine, Cornell University, Ithaca, NY, USA; ^2^ Institute of Biology and Immunology of Reproduction, Bulgarian Academy of Sciences, Sofia, Bulgaria; ^3^ CARI Reproductive Institute, Chicago, IL, USA; ^4^ BioIncept, LLC, Cherry Hill, NJ, USA; ^5^ Society for the Investigation of Early Pregnancy (SIEP), Cherry Hill, NJ, USA; ^6^ Department of Population Health and Reproduction, School of Veterinary Medicine, University of California-Davis, Davis, CA, USA; ^7^ Ross University School of Veterinary Medicine, Basseterre, St. Kitts, West Indies; ^8^ Promigen Life Sciences, Downers Grove, IL, USA; ^9^ Therapeutic Validation Core, Indiana University Simon Cancer Center, Indiana University School of Medicine, Indianapolis, IN, USA

**Keywords:** recurrent pregnancy loss, preImplantation factor (PIF), oxidative stress, PDI, embryo, Autophagy

## Abstract

Recurrent pregnancy loss (RPL) affects 2-3% of couples. Despite a detailed work-up, the etiology is frequently undefined, leading to non-targeted therapy. Viable embryos and placentae express PreImplantation Factor (PIF). Maternal circulating PIF regulates systemic immunity and reduces circulating natural killer cells cytotoxicity in RPL patients. PIF promotes singly cultured embryos’ development while anti-PIF antibody abrogates it. RPL serum induced embryo toxicity is negated by PIF. We report that PIF rescues delayed embryo development caused by <3 kDa RPL serum fraction likely by reducing reactive oxygen species (ROS). We reveal that protein disulfide isomerase/thioredoxin (PDI/TRX) is a prime PIF target in the embryo, rendering it an important ROS scavenger. The 16F16-PDI/TRX inhibitor drastically reduced blastocyst development while exogenous PIF increased >2 fold the number of embryos reaching the blastocyst stage. Mechanistically, PDI-inhibitor preferentially binds covalently to oxidized PDI over its reduced form where PIF avidly binds. PIF by targeting PDI/TRX at a distinct site limits the inhibitor’s pro-oxidative effects. The >3kDa RPL serum increased embryo demise by three-fold, an effect negated by PIF. However, embryo toxicity was not associated with the presence of putative anti-PIF antibodies. Collectively, PIF protects cultured embryos both against ROS, and higher molecular weight toxins. Using PIF for optimizing *in vitro* fertilization embryos development and reducing RPL is warranted.

## INTRODUCTION

In order to effectively develop in culture, embryos depend on autocrine factors [1, 2]. Embryos are also highly vulnerable to reactive oxygen species (ROS) and multiple attempts have been utilized to reduce such vulnerability [3, 4]. Identification of an endogenous compound that would bind specific targets within the embryo to reduce ROS or other toxicity could be of great benefit. There are ongoing concerns that culturing multiple embryos for a prolonged period can lead to epigenetic adverse effects [5–8]. Cases in point are rare imprinting disorders such as Angelman syndrome, Beckwith-Wiedemann syndrome and retinoblastoma in children who are conceived with the use of *in vitro* fertilization (IVF) [9]. Prolonged embryo cultures can also lead to premature delivery [10].

Embryos that remain viable throughout gestation secrete an endogenous compound that promotes self-development and protects against adverse environment. PreImplantation Factor (PIF) is a CD2 associated protein product present in embryos, fetal and placental tissues and in maternal circulation of several mammals [11–16]. In embryo culture media PIF is detectable at the 2-cells stage in mouse, 4-cells stage in humans and by 6-cells in bovine embryos [16, 17]. PIF levels in pregnant circulation just ten days after artificial insemination correlate with live birth [18]. Exogenous administration of synthetic PIF (matches native peptide sequence) targets viable bovine, equine and murine embryos in autocrine and paracrine manner. The effect is not replicated by a control, scrambled PIF [16, 17].

In singly cultured IVF bovine embryos, short-term exogenous PIF promotes blastocyst development [19], while anti-PIF monoclonal antibody has inhibitory effects [16]. PIF promotes endometrial receptivity independent of progesterone and trophoblast invasion [15, 20–23]. To establish embryo/maternal dialogue, PIF binds systemic CD14+ cells (monocytes) and mitogen-activated lymphocytes, reducing proliferation and leading to TH2/TH1 cytokine bias. Interaction with CD3+ T cells increases in maternal circulation during pregnancy, reflecting adaptive response [24, 25]. In non-pregnant pre-clinical models of autoimmunity, transplantation and brain injury, PIF reduced oxidative stress and protein misfolding *via* an integrated local and systemic effect [22, 23, 25–33]. This observation led to the FAST-Track FDA-clinical trial using PIF to treat an autoimmune disease recently completed successfully demonstrating high safety (NCT02239562).

With respect to recurrent pregnancy loss (RPL), PIF reduced circulating NK cells toxicity by decreasing pro-inflammatory CD69 expression [34]. The embryo toxicity assay (ETA) is used clinically to examine RPL serum toxicity, which PIF negates, promoting both development and reducing embryo demise *in vitro* [35, 36]. There is also evidence that certain autoantibodies impair cultured embryo development [36, 37]. PIF's direct protective effects in the embryo and immune cells involve prime targets such as protein-disulfide isomerase (PDI) containing the antioxidant thioredoxin (TRX) domain and heat shock proteins (HSP70 and HSP90) [17] [38]. Both proteins reduce oxidative stress and protein misfolding essential for embryo development [39]. PDI/TRX is involved in oocyte maturation, [40] gamete fusion [41] zona hardening and monospermy [42] as well as proliferation preventing inner cell mass apoptosis [43]. Also, addition of exogenous TRX protects against ROS [7]. However, whether PIF by targeting endogenous PDI/TRX in the embryo can protect against ROS is unknown, though plausible, since PIF protects against ionized radiation [44].

RPL serum is complex, impairing both embryo development and survival [19]. Herein, we examine whether PIF can counteract those distinct phenomena which may be due to ROS. On the other hand, PIF's autotrophic effects on the embryo are negated by added anti-PIF antibody [16]. Moreover, PIF anti-apoptotic action is dependent on the p53 pathways and expression in the placenta is low in intrauterine growth restriction (IUGR) and preeclampsia which may be caused by putative anti-PIF antibodies [45]. Therefore, we examined whether such putative antibodies are present in the RPL sera. We report that PIF protects against ROS supporting its supplementation to protect against epigenetic changes that may occur in long term IVF cultures.

## RESULTS

### PIF acts as a rescue factor, negating embryo toxicity induced by fractioned RPL serum

**Figure 1:**
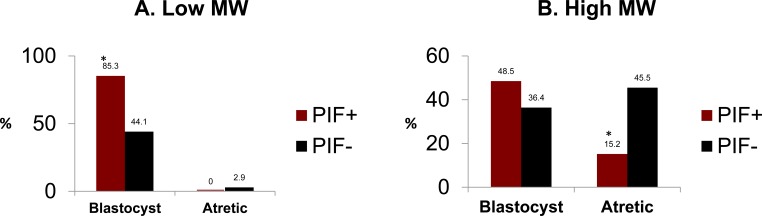
PIF effect on fractionated RPL sera cultured with embryos ETS+ serum was fractionated by Amicon filter to < 3 and > 3 kDa fractions. The effect of these fractions was tested in standard embryo toxicity assay determining the effect of PIF (0.31μg/ml). **A**. In the < 3 kDA fraction, PIF increased the rate of embryos reaching the blastocyst stage. *(Df, 10.8, *p* < 0.009) **B**. In > 3 kDA fraction, PIF decreased the rate of embryos that became atretic. *(Df 7.1, *p* < 0.007). See Supplementary Table 1 for further information.

We previously showed that PIF protects against embryo toxicity by negating the 5% unfractionated RPL sera. [19] To better define toxic factors involved and mechanisms involved in PIF induced protection we therefore separated patients’ with embryo toxic serum (ETS+) to low < 3kDa and higher > 3kDa molecular weight fractions to ascertain whether embryo development was affected differentially. The preliminary with fractionated sera showed that PIF at 0.312μg/ml was most effective in preventing embryo toxicity, and used for testing. The premise was that ROS are low molecular weight species < 3kDa and highly toxic to the embryo. Since PIF protects against oxidative stress, such a separation may capture those toxins effectively. The < 3kDa fraction added to different mouse embryo cultures led to a significant delay in embryo development where only half of the embryos reached to the blastocyst stage. However, by adding PIF to the embryo cultures, up to two fold increase in the blastocyst rate was observed, composite data (85.3% *vs* 44.1%, (Df, 10.8, *p* < 0.009) (Figure 1A). Importantly, no differences were noted in embryo demise rates.

When the > 3kDa fraction was added to embryo cultures the high rate of embryo demise was reversed by the addition of PIF, composite data (15.2% *vs* 45.5% in control, Df 7.1, *p* < 0.007) (Figure 1B) (Supplementary Table 1) Moreover, the number of embryos reaching the blastocyst stage in the PIF treated embryos was slightly higher, 48.5% in PIF treated as compared to control, 36.4% (Df, 5.6, *p* < 0.01). There were also major differences between PIF's effect in < 3kDa fraction reaching high blastocyst rate, 85.3%, as compared with PIF's effect in the > 3kDa fraction reaching only 48.5%. This reveals PIF's major protective effect mostly on embryo demise.

### PIF embryo-protective action against ETS+ does not involve neutralization of anti-PIF antibodies

**Figure 2:**
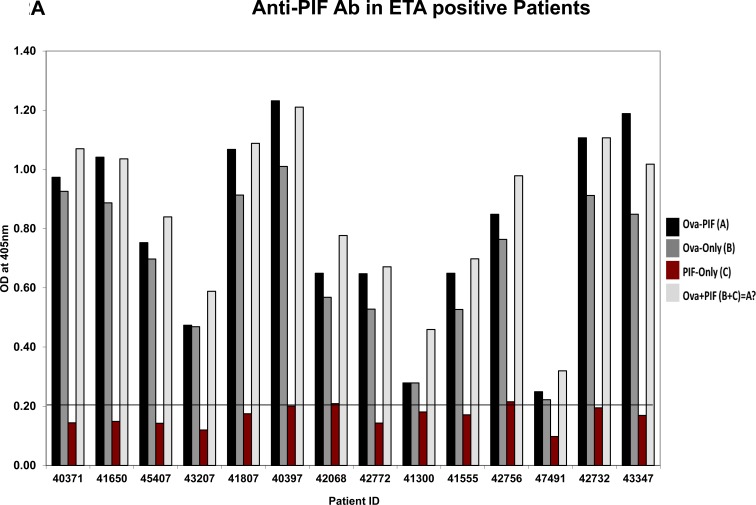

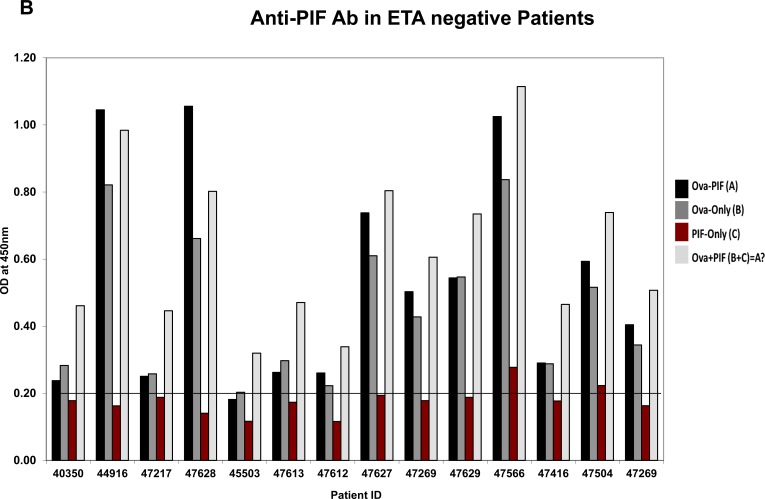
Analysis of anti-PIF antibody presence in RPL serum Twenty eight patients categorized by the ETS assay as ETS+ or ETS- N = 14/group were tested for presence of anti-PIF antibodies. (see methods for assay details). **A**. Anti-PIF Ab in ETA positive Patients, **B**. Anti-PIF Ab in ETA negative Patients. The data showing the lack of anti-PIF antibody in both patient's groups. The presence ova albumin in the assay leads to non-specific binding, but not observed when PIF tested alone. See Table 2 for statistical evaluation.

The > 3KDa fraction of the ETS+ RPL serum mostly increased embryo demise which PIF negated. Such toxicity may involve circulating antibodies [36] including anti-PIF antibodies which inhibited embryo development [19]. Considering that PIF expression is low in high risk pregnancy placentae [23], herein we examined whether this may be related to endogenous anti-PIF antibodies. The embryo-toxicity assay was determined in 28 patients with a history of RPL, determining those with embryo toxic ETS+ or (ETS) sera, control group, [19]. The presence of anti-PIF antibodies were tested in both groups by using a newly developed direct ELISA. To examine anti-PIF-antibody presence each sample was tested in ovalbumin -PIF conjugate, PIF alone or ovalbumin alone coated on the plate were compared to control. As Figure 2A (ETS+ patients) and 2B (ETS- patients) show, in all cases irrespective of ETS status, the PIF coated alone plate had non-detectable anti-PIF-antibody levels in the RPL sera (Supplementary Figures 1-3, assay plate images). In contrast, the ovalbumin alone coated plates showed significant antibody presence likely against egg components. The combined PIF-albumin coated plate did not further potentiate the optical density (OD) as compared with ovalbumin coated alone. However further analysis using a stringent 0.6 OD as cut off showed that higher ETS+ patients 11/14 had anti-Ova antibody *vs* ETS- patients who had only in 4/14 cases (Df 7.0, *p* < 0.007).

### PIF's effect on protein-disulfide isomerase (PDI) inhibitor induced ROS in embryo cultures

**Figure 3:**
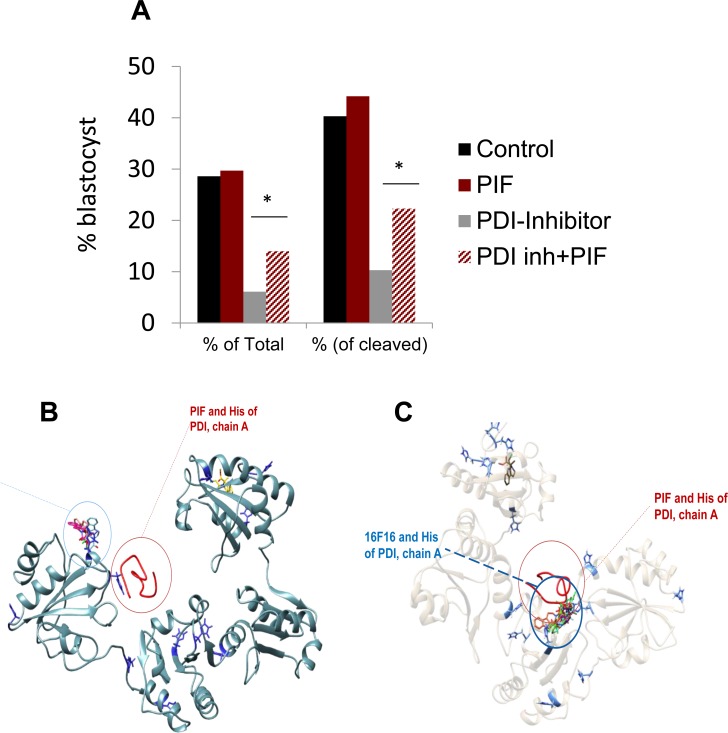
PIF effect on PDI inhibitor induced oxidative stress in cultured embryos Bovine IVF embryos were cultured in the presence of covalent PDI inhibitor. The effect of PIF at ~1:1 ratio was determined after 8 days of culture. PIF increased the number embryos that reached the blastocyst stage both as a percent of the total as well the percent of the cleaved embryos. *(*p* < 0.05). See Supplementary Table 2 for further information. **B**., **C**. PIF and PDI inhibitor (16F16) interaction with PDI. PIF binds more avidly to reduced PDI, forming higher number of interactions to His, but also highest electrostatic potential based interaction energies compared to 16F16. **B**. Comparative docking of PIF and 16F16 to PDI structural models of oxidized PDI. **C**. Same docking to reduced form of PDI. PIF binding was predicted using flexible peptide docking algorithm FlexPepDock, while small molecule inhibitor 16F16 binding was predicted using AutoDock Vina semi-rigid docking. *Histidine* residues are depicted in blue, PIF is depicted in red, 16F16 conformers are depicted in different colors.

The above data showed that the < 3kDa fraction induced delay in embryo development was negated by PIF, while not affecting embryo survival. Whether the stunted embryo development is due to ROS was further examined. This is due to the fact that PIF's prime targets in the embryo are PDI/TRX proteins which are known to reduce oxidative stress [17, 19]. The effect of a covalent PDI inhibitor was examined in bovine embryos cultured in groups (Figure 3A) (Supplementary Table 2). The PDI inhibitor 16F16 added alone reduced the cleavage rate as compared to the control group, media culture only (*p* = 0.005), but was not different from the other groups. Addition of the PDI inhibitor reduced the number of embryos developing to the blastocyst stage, whether as a proportion of the total number of oocytes or as a proportion of cleaved zygotes (*p* < 0.0001). Logistic regression indicated that the interaction of 16F16 and PIF was significant (*p* = 0.02). Further analysis confirmed that this was because PIF alone had no significant effect (*p* = 0.85), but the detrimental effect of 16F16 was partially reversed by inclusion of PIF in the culture medium. Blastocyst rate was significantly different for 16F16 *versus* 16F16 plus PIF (*p* = 0.0056 considering total oocytes included and *p* = 0.0092 considering only cleaved zygotes). The total blastocyst formation in control was 40% which was reduced four-fold by the PDI inhibitor. Importantly PIF increased this rate over two fold. PIF supplementation alone did not change the rate of embryo development to blastocyst stage relative to the control group (*p* = 0.82).

### PIF reduces ROS by avidly targeting reduced PDI at a site distinct from the covalent PDI inhibitor- *in silico* design

The increase in ROS by the PDI/TRX inhibitor delayed embryo development which PIF reduced, similar to the < 3kDa fractionated serum. Possible protective mechanisms involved in PIF's action [17] are exerted by docking with a higher energy to oxidized PDI. Using a frequently employed small molecule docking algorithm of Vina, several docking places of the small molecule inhibitor of PDI, 16F16 were found when docking models of PIF and 16F16 were compared for oxidized (Figure 3B) and reduced forms (Figure 3C) of PDI. Crystallography models of human PDI indicated that PIF binds closely to one of the most frequently predicted (based on number of models) sites of 16F16 in reduced PDI.

PIF was found (by FlexPepDock) to be bound at the same site as the top scored Vina docking predicted 16F16 model. Most of the other models indicated binding at the same place (Figure 3C).

**Figure 4:**
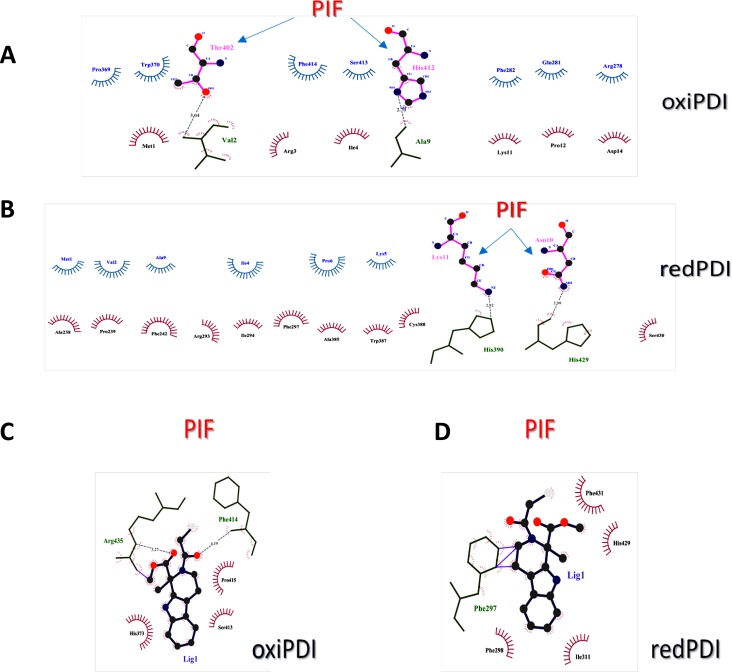
Analysis of PIF and PDI inhibitor interaction with PDI **A**. Schematic diagram of oxidized PDI-PIF interactions generated by LigPlot+. **B**. Schematic diagram of reduced PDI-PIF interactions generated by LigPlot+. **C**. Schematic diagram of oxidized PDI-16F16 interactions generated by LigPlot+. **D**. Schematic diagram of reduced PDI-PIF interactions generated by LigPlot+. Hydrogen bonds are indicated by dashed lines between the atoms involved, while hydrophobic contacts are represented by an arc with spokes radiating towards the ligand atoms they contact. The contacted ligand atoms are shown with spokes radiating back.

Using LigPlot+ [46] donor-acceptor and hydrophobic interactions were depicted in the binding interfaces of oxidized and reduced forms of PDI when bound by either PIF or 16F16. A higher number of hydrophobic interactions in favor of the reduced PDI was found when bound to PIF (Figure 4B), compared to the oxidized form of PDI (Figure 4A). Only one His (His412) participated in the PIF binding interface of the PIF-oxiPDI complex, while two His (His390, His429) were engaged in PIF-redPDI complex.

The specific PDI small-molecule inhibitor 16F16 was found to act as a thiol alkylating agent, where a highly electrophilic moiety forms a covalent bond with the PDI active site to prevent further reductase activity [47]. Since 16F16 inhibitory activity against PDI is driven not by binding affinity but by the redox potential of their reactive moieties [48], we focused on the role of the PDI active site. PDI is but one member of a large superfamily of thioredoxin proteins that mediate the reduction, oxidation and isomerization of disulfide bonds on other proteins [49, 50]. The N-terminal active site cysteine of the domain of PDI participating in disulfide bond isomerization is stabilized as a thiolate anion by the pKa of the local histidine imidazole [51].

Since 16F16 was found to bind His residues in some of its known targets [48] we followed His pattern of binding towards PDI as well. It (F16F) was bound to only one His in both oxiPDI (His373) and redPDI (His429), but other close by residues that were bound by 16F16 in oxiPDI-16F16 complex like Ser413, Phe414, were also bound by PIF (Figure 4C). Interestingly, only Phe297 was shared by 16F16 and PIF in redPDI complex (Figure 4D).

### Binding similarities of PIF and 16F16 to PDI

To explain the similarity in reduced PDI binding pockets of PIF and 16F16, some binding/interaction/solvation energies of 16F16/PIF and oxi/red PDI (Supplementary Table 3) were estimated.

PIF had the highest electrostatic potential based interaction energies (APBS binding E, Electrostatic E, Electrostatic Interaction, MEP interaction E) to redPDI, superseding 16F16. This resulted in increased solvation energy of PIF as well in reduced form of PDI when compared to its oxidized form. PIF, compared to 16F16 in its docking to redPDI, demonstrates better energetic binding properties. This corroborated the LigPlot+ scheme of pocket binding where 16F16 was found to have several ligand bonds of non-polar and hydrophobic interactions, while PIF had more hydrophobic interactions and two hydrogen bonds. In contrast, in oxiPDI complex 16F16 had the same number hydrogen bonds as PIF.

Based on MEP interaction energy and Electrostatic energy profiles of binding, we conclude that 16F16 binds more avidly to oxiPDI than to redPDI, and that PIF is even more strongly bound to redPDI than 16F16 is. This could explain its ability to at least partially abrogate 16F16 effect at a roughly one to one molar ratio of PIF and inhibitor. Thus, PIF, through targeting at a distinct site, has the ability to minimize the PDI inhibitor action.

**Figure 5:**
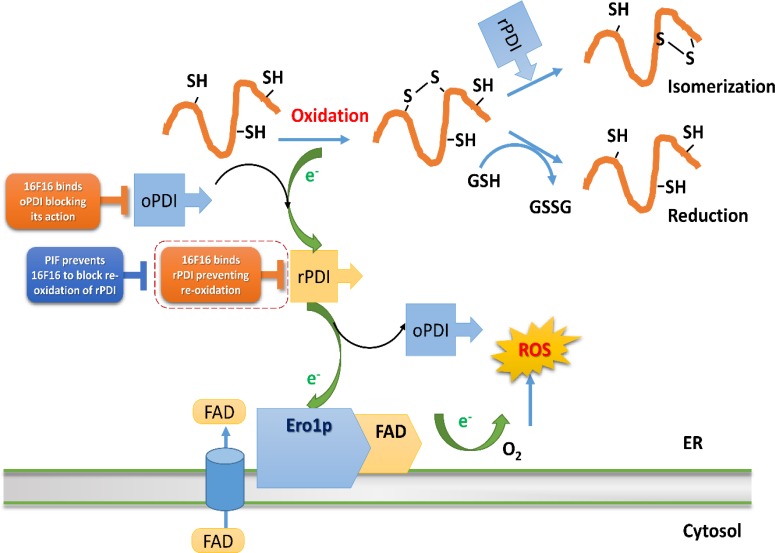
PIF mechanism of action on PDI oxi/reduction pathways Small molecular inhibitor 16F16 and PIF act differently on oxidative protein folding exerted by PDI. The scheme is adopted by “Biochemical Basis of Oxidative Protein Folding in the Endoplasmic Reticulum” by Benjamin P. Tu et al. [66]. Protein disulfide isomerase (PDI), has two thioredoxin-like active sites, which both contain two cysteine residues and it is responsible for the formation of the disulfide bonds in unfolded eukaryotic proteins. By transferring the disulfide bond between these two cysteine residues onto the folding protein it is responsible for the folding protein oxidation, becoming reduced one (rPDI). Then PDI must be reoxidized so it can further catalyze the formation of disulphide bonds in unfolded proteins. This is carried out by an endoplasmic reticulum (ER) membrane-associated protein, Ero1p, which forms a mixed disulfide with PDI, which is resolved by a nucleophilic attack of the second cysteine residue in one of the active sites of PDI. As a result, oxidized PDI (oPDI) is obtained. Ero1p itself is oxidized by transferring electrons to molecular oxygen. As it is an FAD-binding protein, this transfer of electrons is strongly favored when Ero1p is bound to FAD. In misfolded proteins, incorrect disulfide bonds are rearranged by reduction, which is carried out by oxidation of reduced glutathione (GSH) to oxidized glutathione (GSSG). 16F16 and PIF predominant binding mode of action shown. ER, endoplasmic reticulum; PDI, Protein disulfide isomerase; rPDI, reduced PDI; oPDI, oxidized PDI.

PIF interference with 16F16 small molecule inhibitor is considered in the context of PDI oxidative folding, suggesting that different modes of action of PIF and 16F16 allow for the observed phenomenon (Figure 5). PIF's stronger binding with reduced PDI abolished 16F16 action on oxidized PDI, hence PDI re-oxidation was re-established.

## DISCUSSION

The major finding herein is that ROS likely present in < 3kDa RPL serum delays cultured embryos development which PIF prevents. Mechanistically, pro-oxidative PDI covalent inhibitor induced delay is reduced by PIF, further substantiating such premise. PIF targets PDI/TRX in the embryo when the enzyme is in its reduced form and binds at a distinct site, impairing covalent PDI inhibitor binding leading to ROS formation. In the > 3kDa RPL sera fraction, PIF reduced embryo demise and increased blastocyst rate, which is not exerted through neutralization of anti-PIF antibodies. Thus the low expression of PIF in the placenta in high risk pregnancy is not due to the presence of circulating putative anti-PIF antibodies. [45]

Currently the serum for embryo toxicity is analyzed as a whole, without separation [52–55]. Herein we show for the first time major differences between low and higher molecular weight ETS+ effects and associated PIF-induced protection. In the < 3kDa fraction PIF prevented a delay in development possibly due to ROS. Singly cultured bovine embryos secrete and are targeted by PIF, to promote development confirmed herein [16, 17, 19]. The covalent PDI inhibitor binds PDI/TRX —a prime PIF target— severely impairs embryo development [17]. Mild effect in cleavage was coupled with four-fold decrease in blastocyst rates, substantiating the PDI/TRX critical role. [17]. PDI inhibitor (16F16) at > 3.125uM dose led to total arrest of embryo development, while other cells were impaired at different concentrations [44]. Another PDI inhibitor, bacitracin, already damaged oocyte maturation at 5 μM concentration [35]. Thioredoxin added to pronuclear stage embryos released 2 cell block and promoted embryos development [7, 56]. PIF targeted the endogenous PDI/TRX suggesting an autotrophic loop, supporting its critical role [16]. Beyond targeting PDI/TRX, by binding HSPs and Kv1.3b K^+^ channel, PIF may further amplify the protective activity [17, 32]. Collectively, both the < 3 kDa ETS+ fraction and the PDI inhibitor delayed embryo development likely exerted through ROS formation which PIF fully in the former and partially in the latter reversed. This is critical for IVF success.

PIF as single administration more than doubled the blastocyst rate despite the low molar PIF to PDI inhibitor ~1:1 ratio and the enriched specific SOF-BE1 media containing Myoinositol (an antioxidant), and insulin [57]. Insulin may interfere with PIF activity since the peptide targets the insulin degrading enzyme in the embryo [17]. Failing embryos may release lysosomes and peptidases, likely to degrade PIF in long term culture [18]. SOF-BE1 media is optimal, therefore lack of PIF effect was expected in contrast to that observed in singly cultured embryos [35].

The PDIs are enzymes that share similar functions mostly acting through thioredoxin. PDI/TRX interacts with HSPs, ubiquitin, serpins and importantly CD9, involved in sperm/egg interaction which may also be impaired indirectly following exposure to 16F16 (String analysis). PDI mechanism is expected to be complex and the depiction is representative (Figure 5). In order to function, the endogenous enzyme needs to be re-oxidized to enable protein folding. The reduced (dithiol) form of PDI is able to catalyze a reduction of mis-paired thiol residues of a particular substrate, also acting as an isomerase. oxiPDI is more likely to bind to 16F16.

During the folding process, PDI becomes reduced, obtaining electrons from the protein SH-groups, thus creating a disulfide bond. In order to get re-oxidized, PDI has to donate its electrons to Ero1p, which in turn binds to FAD protein. Unlike bacteria, where this is coupled to the respiratory chain, in eukaryotic cells the electrons are transferred to molecular oxygen, possibly causing excessive ROS. Human hPDI, (abb’xa’) is in both reduced and oxidized states, and has four thioredoxin domains, arranged in a horseshoe shape with two CGHC active sites. In reduced hPDI, domains a, b, and b’ line up in the same plane, whereas domain a’ twists ~45° out. In oxidized hPDI, the four domains are organized differently to stay in the same plane, and the distance between the active sites increases. In contrast to the closed conformation of reduced hPDI, oxidized hPDI exists in an open state, with more exposed hydrophobic areas and a larger cleft with potential for substrate binding [58]. Sterically, PIF could have less effect on oxiPDI; this form of PDI is involved in protein folding but not in electron exchange. Hence, the redPDI is less likely to produce ROS. In the absence of PIF, 16F16 would likely bind to both PDI forms and impair both accepting and donating electrons. Thus, 16F16 binding predominantly to oxiPDI could cause excessive ROS and lead to embryo damage. [59, 60] PIF, on the other hand, is able to block redPDI, most likely by impairing 16F16 binding. This prevents redPDI from transitioning to oxiPDI, a step where excessive electrons, ROS, are produced. At higher PIF (5-10 fold) concentration, the binding to PDI could drive the inhibitor out from its pocket, thus rendering PDI in a favorable, reduced state. Such data support the view that PIF action is target-specific.

Interestingly, the > 3kDa RPL serum three-fold increase in embryo demise and mildly reduced embryo development was negated by PIF. Thus, in RPL PIF can negate disparate toxic elements and NK cells cytotoxicity supporting therapeutic potential [34]. RPL serum contains several embryo-toxic compounds and antibodies [36, 61]. Consequently, they are used in patients’ workup and possibly as targets for therapy. We show that PIF-induced protective action on the embryo does not involve neutralization of putative anti-PIF antibodies examined in both ETS+ and ETS- samples. The anti-ovalbumin antibody concentration varied significantly from non-detectable to high levels irrespective of whether it was from ETS+ or ETS- samples, indicating a lack of involvement in the embryo toxic activity. Thus planned therapeutic application of PIF in RPL and for high risk pregnancy treatment is unlikely to be negated by the presence of those antibodies.

Overall, PIF, a human embryo derived compound, made as synthetic PIF GMP quality, may be used to supplement IVF culture media to prevent potential embryo toxicity that develops in prolonged and multiple embryo cultures.

Using fractionated sera is a new approach, enabling to identify toxic effects which can translate to adverse pregnancy events post-implantation. Further fractionation could reveal rapidly and non-invasively critical elements involved in embryo wellbeing and may support appropriate intervention starting prior to pregnancy.

In conclusion, PIF promotes both embryo development and survival by negating ETS+ effect. PIF's protective effect is dependent on targeting PDI by reducing ROS of cultured embryos. Both murine and bovine embryos were protected by PIF which supports its clinical application also to human IVF. PIF completed successfully phase I clinical trial for autoimmune disease (NCT02239562). The results herein support PIF's translation for targeted RPL therapy as well.

## MATERIALS AND METHODS

### Synthetic preImplantation factor (PIF) and PDI inhibitor

PIF (MVRIKPGSANKPSDD) was synthesized by solid phase peptide synthesis (Peptide Synthesizer, Applied Biosystems) employing Fmoc (9-flourenylmethoxycarbonyl) chemistry at Bio-Synthesis (Lewisville, TX). Final purification was carried out by reversed-phase HPLC and identity was verified by matrix-assisted laser desorption/ionization time-of-flight mass spectrometry and amino acid analysis at > 95 % purity. 16F16, a covalent PDI inhibitor, was purchased from Sigma Aldrich (St. Louis, MO).

### PIF effect on mouse embryos cultured with fractionated embryo-toxic serum (ETS+)

The study was approved by the CARI Research Institute, Chicago, IL. Archived frozen serum samples from patients with recurrent pregnancy loss of various etiologies were studied. Serum samples which were previously found to be toxic for cultured mouse embryos (ETS+) determined by using established criteria as previously reported [19, 36] was chosen. PIF promotes cultured blastocysts development, and in RPL unfractionated sera, PIF both prevented a delay in development and reduced embryo demise [19]. The effect is also specific since scrambled PIF (same sequence but in a random order, used as a control) was not up-taken by cultured mice embryos [16]. Our previous report showed that under optimal conditions the rate of mouse embryos reaching the blastocyst stage is above 80% [19].

Embryo Toxicity Assay was performed as previously reported. [19] These sera were used for the anti-PIF antibody (see below) as well for the serum fractionation. Briefly, two-cell stage embryos (N = 134) were collected from super ovulated mated CB6F1/J mice. Removed oviducts were dissected under microscope and embryos collected were transferred into mHTF media (Cat# 2001, InVitroCare, Frederick, MD). To better understand the etiology of embryotoxicity the serum samples were separated into low and high molecular weight fractions using a filter system. Serum filtration was performed using a < 3 kDa Amicon Ultra-4 system following manufacturer's protocol. Collected tubes were spun at 4000 x g for 20min using 3kDa filter. The resulting < 3kDa filtrate and > 3kDa serum proteins were collected. The separated < 3kDa and > 3kDa serum fractions at 5% were added to randomly partitioned 2-cell mouse embryos with or without (48-6250 ng/ml) PIF as a screening experiments. Based on the preliminary data generated the 0.312 μg/ml PIF concentration was chosen as most effective and was tested at least 5 embryos (per experiment, patient < 3 kDa and > 3kDa groups). The culture media added 5% fractionated sera were adjusted to a total to 500μl culture volume with BSA [19]. Embryos were cultured in Nunc Petri dish in 500μl of culture medium HTF HEPES (cat# 2001, InVitroCare, Frederick, MD) in the presence of 5% heat inactivated patients’ < 3kDa or > 3kDa sera under mineral oil by incubating at 37°C with 5% CO_2_ for 3 days maintaining pH 7.2 throughout the experiment as previously shown [19]. At the end of the culture period at 72 hours, embryos developmental stages were recorded using established morphological criteria by using a microscope. Embryos were categorized as blastocysts, pre-blastocysts, morula, number of cells, 6-8, 2-4 or atretic embryos, and evaluated by two different observers (CWS, RGR).

### ELISA to detect anti-PIF antibody in human serum

Anti-PIF antibody detection was carried out in 28 patients with a history of RPL having archived sera known to be ETS+ or ETS- based embryo toxicity assay. For such identification an indirect enzyme-linked immunosorbent assay for qualitative determination of anti-PIF antibody was developed. The 10X Ultracoat II, coating buffer (Leinco Technologies, USA) was diluted to 1X with water just before use, and used to dilute dextran-PIF or ovalbumin-PIF or ovalbumin alone to 100ng/ml final concentration and dispensed 100μl per well Nunc Maxisorp plate. Subsequently, the plate was sealed and incubated at 4°C overnight. Following incubation, the coating solution was discarded and washed with PBST, 4 times in a plate washer. 300μl of SEA BLOCK, (Fisher Scientific, Cat. No.0037527) blocking buffer was added to each well and incubated for one hour at 37C. Subsequently the plate was washed with PBST for 4 times and dried. The serum samples were diluted to 1:100 with PBST (1 part sera: 99 part PBST) running the samples in triplicate.

The 100 μl of the diluted serum samples were added to the pre-coated plates coated with PIF or ovalbumin-PIF or ovalbumin alone and incubated at 37Cfor one hour. After plates were washed 4 times using PBST (0.1% Tween 20) and dried. Goat α-human IgG conjugated to alkaline phosphatase, (Roche, Product No. 03-118-495-001) in1% Fish skin gelatin (FSG) in PBS was diluted to 1:2000 of this 100 μl of conjugate was added to the plate and incubated at 37°C for 30 minutes. Subsequently, the plate was washed and 100μl of pNPP substrate was added, incubated for 10 minutes in dark, at room temperature. Reaction was stopped by adding NaOH and the plate was read at 405 nm. The diluted anti-PIF monoclonal antibody was used as a positive control, when using the appropriate secondary antibody. In addition, a secondary antibody control and blank control were used. As control for PIF coat, anti-PIF antibody conjugated to biotin was added to six wells and detected by adding ultravidin HRP. TMB substrate was added to all six wells, but stop solution was added only to three wells, so as to differentiate blue (no stop) and yellow (stopped). The intensity of the signal is directly proportional to the concentration of anti-PIF antibody in the sample.

### *In vitro* production of bovine embryo

Bovine ovaries were obtained immediately after slaughter and transported in warmed (37°C) sterile saline inside insulated containers to the laboratory at Cornell University as previously described [17]. Briefly, the ovaries were rinsed in warmed sterile saline solution in the laboratory and then allowed to cool gradually to approximately 25°C by the end of the oocyte retrieval procedure. All visible follicles between 2 and 8 mm in diameter were aspirated with 18g hypodermic needles using a vacuum pump set so that 22.5-25 mL of fluid was aspirated per minute. Follicular fluid was aspirated into 50 mL conical tubes up to 25 mL per tube. The tubes were allowed to stand for at least 15 min before the sedimented “pellet” was carefully removed and transferred to a 10 cm Petri dish containing holding medium. Cumulus-oocyte complexes (COCs) were then sorted and selected based on visual appearance; only those with homogenous cytoplasm, non-expanded cumulus, and with at least 3 layers of cumulus cells surrounding the oocyte were selected for transfer to the maturation medium. Once identified and removed, the COCs were washed in holding medium and transferred into maturation medium.

Recovered COCs were matured in groups of 10-30 for 24 hrs in TCM-199 (with Earle's Salts) enriched with 10 % FCS, 0.2 mM sodium pyruvate, 1 mM alanyl-glutamine, 0.1 mM taurine, 0.1 mM cysteamine, 1 μg/ml estradiol, 85 mU/ml bovine follicle stimulating hormone (FSH, SIOUX Biochemical, Inc., Sioux Center, IA), 0.1 μg/ml gentamicin, and 10 ng/ml epidermal growth factor (EGF) at pH 7.35 ± 0.02 and osmolarity of 300 ± 2 mOsm and covered with light mineral oil in a humidified atmosphere at 38.5°C with 5 % CO_2_ in air. Matured oocytes were transferred to a modified IVF medium (Fert-TALP; [61]) supplemented with 0.5 mM fructose, 0.2 mM non-essential amino acids, 6 mg/ml BSAFFA Fraction V, 30 μM penicilamine, 15 μM hypotaurine, 1.5 μM epinephrine (PHE), 22 μg/ml heparin, 20 μg/ml gentamicin, covered with light mineral oil in a humidified atmosphere at 38.5°C with 5 % CO_2_ in air for 18 h (pH of 7.38 ± 0.01, 285 ± 1 mOsm). The IVF procedure for bovine species was recently reported. [16, 19] Frozen semen straws from the same two bulls were combined for each experiment. The straws were each thawed at 37°C for 2 min and then combined prior to centrifugation. Sperm were selected by double density gradient (90% and 45% BoviPure^®^; Nidakon, Sweden, distributed by Spectrum Technologies, Healdsburg CA) centrifugation at 400x g for 20 min in centrifuge buckets warmed to 37°C. Subsequently, sperm were washed in 5 ml of BoviWash (Nidakon, Sweden, distributed by Spectrum Technologies, Healdsburg CA) at 400 x g for 5 min and added to oocytes in fertilization medium (SOF-FERT) at a final concentration of 1.5 × 10^6^ sperm/ml and cultured for 18 hrs at 38.5°C with 5 % CO_2_ in air.

After vortexing to remove cumulus cells, presumptive zygotes were transferred in groups of 25 to 50 per wells of a 4-well plate each containing 400 μL of medium (appropriate for each group) overlaid with 300μL of mineral oil and preconditioned by 24 hrs exposure to incubator conditions (38.5°C, humidified atmosphere of 5 % CO_2_, 7 % O_2_ in Nitrogen) [62]. The medium used was “synthetic oviduct fluid - bovine embryo 1” (SOF-BE1) [63,64].

### Effect of PDI inhibitor (16F16) and PIF on cultured embryos

Short term exposure of PIF to singly cultured bovine embryos post-fertilization promoted their development up to the blastocyst stage after exposure for only three days followed by four days of observation. [19] To determine the effect of PIF on cultured embryos development where herein cultured in large groups alone and in the presence of the PDI inhibitor, four experimental groups were examined: control (SOF-BE1 medium), PIF supplemented (5μM) medium, 16F16 (a PDI inhibitor) supplemented (3.125μM) in medium, and the combination of 16F16 and PIF at these concentrations. Experimental conditions were applied after fertilization and cumulus cell removal. Preliminary experiments were conducted to identify a concentration of PDI inhibitor that would only partially inhibit embryo development. Eventually, a concentration of 3.125 μM was used. All higher concentrations (> 3.125μM) completely inhibited embryo development (data not shown).

### PDI binding by PIF and 16F16 - an *in silico* docking analysis to establish binding site and potential mechanism of action

#### Modeling *in silico* of PIF docking to PDI

PIF docking to PDI was examined following the *in silico* flow as previously described [17, 25]. Briefly, a PIF model was generated using *de novo* peptide structure prediction, based on the primary structure (amino-acid sequence) alone [46,47] The top scored models generated by the server PEP-FOLD (http://bioserv.rpbs.univ-paris-diderot.fr/PEP-FOLD/) were used further in docking simulations. The potential binding of PIF to PDI surfaces was assessed in terms of probability, PIF participating residues, targeted protein ligand-receptor surface determining residues by using the PepSite 2 server (http://pepsite2.russelllab.org/). High-resolution peptide docking of PIF to the oxidized and reduced forms of PDI (obtained as PDBs 4EL1 and 4EKZ) was done using Rosetta FlexPepDock server (http://flexpepdock.furmanlab.cs.huji.ac.il/). A PDB encoded model of the *de novo* PIF model was supplied in close proximity and its location was specified by the PepSite 2. FlexPepDock allows full flexibility to the peptide and side-chain flexibility to the target protein, thus providing accurate refinement of the peptide structure, starting from up to 5.5 RMSD of the native conformation. (RMSD - root-mean-square deviation, is the measure of the average distance between the atoms [usually the backbone atoms] of superimposed proteins) [47]. The PIF binding scores to models obtained by crystallography of the reduced and oxidized forms of PDI were previously estimated and published in Barnea et al [17].

#### Modeling *in silico* of PDI inhibitor small molecule 16F16 binding to PDI

16F16 molecular structure (obtained as SDF file) and oxidized and reduced forms of PDI (obtained as PDBs 4EL1 and 4EKZ) were prepared (VEGA ZZ, UCSD Chimera PDB2PQR) as model files suitable for virtual docking using AutoDock Vina (http://vina.scripps.edu). Both were subject to unbiased ligand-receptor docking in full molecular space with no restraints. The high probability models were selected for further analysis as two chain PDB models. Such data provides specific information on the PDI inhibitor binding with PDI.

### Comparison of PIF and 16F16 binding sites on PDI in order to determine PIF inhibitory action

In order to assess potential binding sites, 16F16 and PIF were superimposed using their predicted docking positions over PDI and Chimera as molecular visualization tool. PIF had only one predicted site based on the site predicted by PepSite 2 and refined in its binding by FlexPepDock, while 16F16 had several binding locations predicted by AutoDock Vina, depending on the PDI form - reduced or oxidized.

### Confirmation of 16F16 / PIF ligand-receptor binding to PDI

To assess the ligand-receptor binding interface of 16F16 to PDI and that of PIF to PDI, LigPlot plus algorithm was used to generate automatic schematic diagrams of protein-ligand interactions, based on hydrogen bonds and hydrophobic contacts. Hydrogen bonds are indicated by dashed lines between the atoms involved, while hydrophobic contacts are represented by an arc with spikes radiating towards the ligand atoms they contact. The contacted atoms are shown with spikes radiating back. LIGPLOT algorithm is described in detail in Wallace et al [46]. The program is suitable for both small molecules - protein and peptide/protein-protein interactions.

### Statistical analysis

The effect of PIF on embryo development following exposure to fractionated sera was determined by using chi square analysis and degree of freedom where *p* < 0.05 was considered as statistically significant.

The effect of added PIF and 16F16 on the cleavage of zygotes, assessed at 2 days after fertilization and development of embryos to the blastocyst stage by day 8 after fertilization was compared between groups by mixed-effects logistic regression. Main effects were PIF, PDI-inhibitor, and their interaction. Replicate (week of experiment) was included as a random variable (Stat-IC 11.2 for Windows; StataCorp LP, College Station TX). Where logistic regression indicated overall differences, post-hoc comparisons of selected groups were made using Chi-square statistics (MedCalc Statistical Software version 14.12.0, Ostend, Belgium; http://www.medcalc.org; 2014). *p <* 0.05 was regarded as significant.

## SUPPLEMENTARY MATERIALS FIGURES AND TABLES


