# Correction: PreImplantation factor (PIF) protects cultured embryos against oxidative stress: relevance for recurrent pregnancy loss (RPL) therapy

**DOI:** 10.18632/oncotarget.26505

**Published:** 2018-12-18

**Authors:** Lindsay F. Goodale, Soren Hayrabedyan, Krassimira Todorova, Roumen Roussev, Sivakumar Ramu, Christopher Stamatkin, Carolyn B. Coulam, Eytan R. Barnea, Robert O. Gilbert

**Affiliations:** ^1^ Department of Clinical Sciences, College of Veterinary Medicine, Cornell University, Ithaca, NY, USA; ^2^ Institute of Biology and Immunology of Reproduction, Bulgarian Academy of Sciences, Sofia, Bulgaria; ^3^ CARI Reproductive Institute, Chicago, IL, USA; ^4^ BioIncept, LLC, Cherry Hill, NJ, USA; ^5^ Society for the Investigation of Early Pregnancy (SIEP), Cherry Hill, NJ, USA; ^6^ Department of Population Health and Reproduction, School of Veterinary Medicine, University of California-Davis, Davis, CA, USA; ^7^ Ross University School of Veterinary Medicine, Basseterre, St. Kitts, West Indies; ^8^ Promigen Life Sciences, Downers Grove, IL, USA; ^9^ Therapeutic Validation Core, Indiana University Simon Cancer Center, Indiana University School of Medicine, Indianapolis, IN, USA

**This article has been corrected:** The correct Author name is given below:

**Soren Hayrabedyan**

Original article: Oncotarget. 2017; 8:32419-32432. https://doi.org/10.18632/oncotarget.16028

