# Supplementary material for: PreImplantation factor (PIF) protects cultured embryos against oxidative stress: relevance for recurrent pregnancy loss (RPL) therapy
Source: Oncotarget. 2017 Mar 8;8(20):32419–32. doi: 10.18632/oncotarget.16028 (PMC5464799; doi:10.18632/oncotarget.16028)
Supplement: Supplementary file 1 [file oncotarget-08-32419-s001.pdf]

PreImplantation factor (PIF) protects cultured embryos against oxidative stress: relevance for recurrent pregnancy loss (RPL) therapy

Supplementary Material

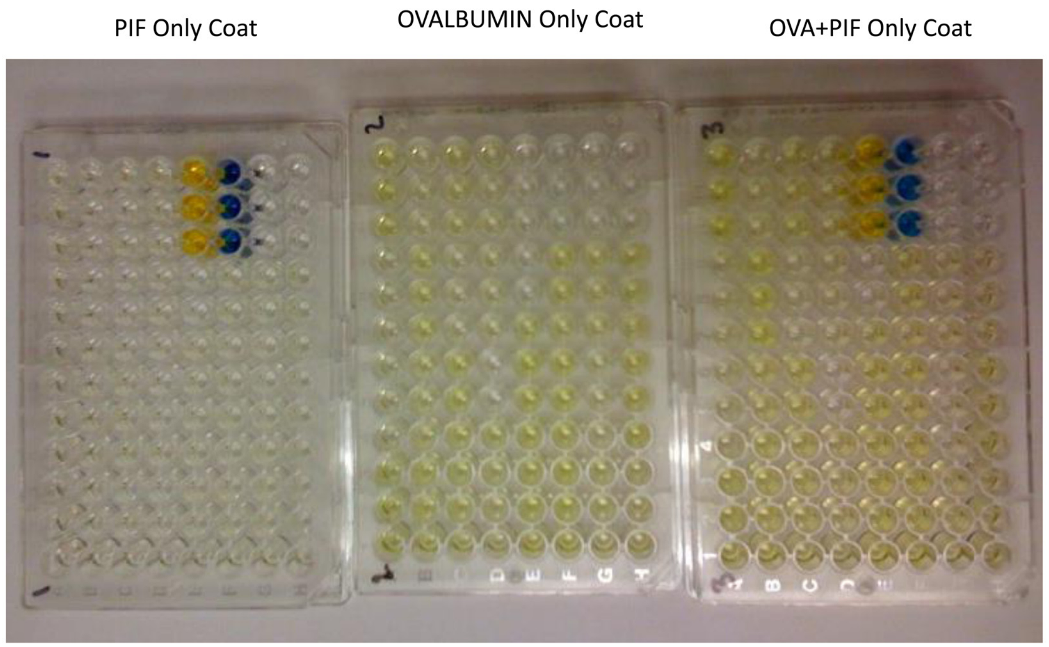

Supplementary Figure 1: ELISA based PIF antibody detection: data shows that the antibody detected is likely against egg component and not against PIF

OVA+PIF Only Coat

OVALBUMIN Only Coat

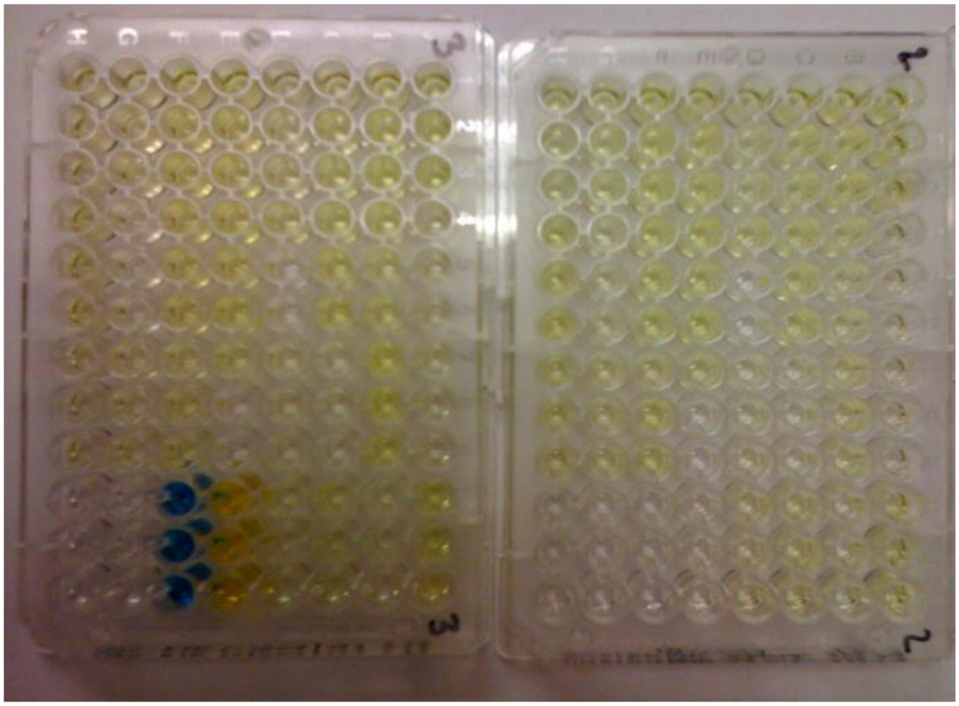

Supplementary Figure 2: ELISA based PIF antibody detection: data shows that the antibody detected is likely against egg component and not against PIF

PIF Only Coat

OVALBUMIN Only Coat

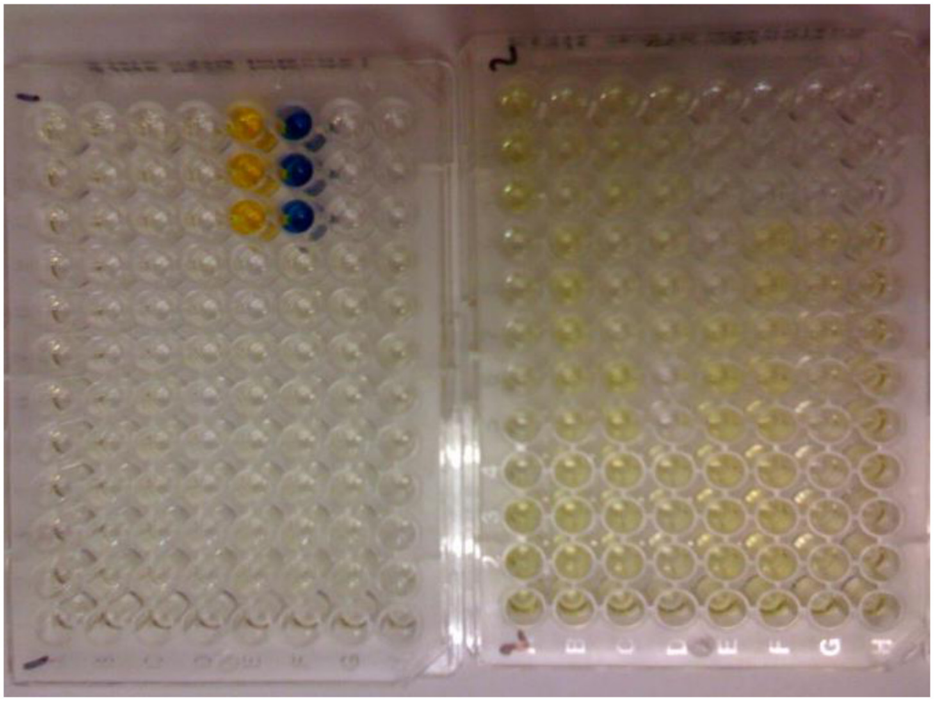

Supplementary Figure 3: ELISA based PIF antibody detection: confirmation that only the ovalbumin presence and not PIF leads to the antibody detection.

Supplementary Table 1: PIF PREVENTS EMBRYO DEMISE CAUSED BY RPL SERA
<3kDa Fraction Causes Embryo Delay & >3kDa Fraction Causes Embryo Demise

**PIF+**	34 Total	**PIF-**	34 Total
P29 Blast	85.3%	15 Blast	44.1%
2 Eblast	5.9%	4 Eblast	11.8%
1 Morula	2.9%	8 Morula	23.5%
0 6-8 Cell	0.0%	3 6-8 Cell	8.8%
2 2-4 Cell	5.9%	3 2-4 Cell	8.8%
0 Atretic	0.0%	1 Atretic	2.9%

< 3 kDa sera fraction

**PIF+**	33 Total	**PIF-**	33 Total
16 Blast	48.5%	12 Blast	36.4%
5 Eblast	15.2%	2 Eblast	6.1%
6 Morula	18.2%	4 Morula	12.1%
0 6-8 Cell	0.0%	0 6-8 Cell	0.0%
1 2-4 Cell	3.0%	0 2-4 Cell	0.0%
5 Atretic	15.2%	15 Atretic	45.5%

> 3 kDa sera fraction

Supplementary Table 2: PIF PREVENTS EMBRYO DEMISE CAUSED BY OXIDATIVE STRESS Blocks PDI / Thioredoxin Inhibitor Effect

Group		Cleaved		Blastocyst		
		N	%	N	% (of total)	% (of cleaved)
Control	248	176	70.0^{a}	71	28.6^{a}	40.3^{a}
**PIF**	**155**	**104**	67.1^{ab}	**46**	29.7^{a}	44.2^{a}
PDI-inhibitor	247	145	58.7^{b}	15	6.1^{b}	10.3^{b}
**PDI inh+PIF**	**236**	**148**	60.1^{ab}	**33**	14.0^{c}	22.3^{c}

PIF effect on cultured bovine zygotes cleavage and reaching the blastocyst stage.
($P < 0.05$). N = No. of Embryos
Total zygote cleavage and embryo development to blastocyst stage for each treatment group.
(Replicates are combined.) Values in the same column with different superscripts are significantly different ($p < 0.05$).

Supplementary Table 3. Ligand-receptor interaction analysis in docking models of PIF and 16F16 to either oxidized PDI or reduced PDI form [1].

	oxi PDI - PIF	oxi PDI - 16F16	red PDI - PIF	red PDI - 16F16
APBS Binding energy [kJ/mol]	-4.07	5.95	**-6.95**	0.40
Electrostatic Interaction energies [kcal/mol]	9.43	-13.42	**-18.47**	3.00
Electrostatic Interaction energies (distance dependent) [kcal/mol]	-5.46	-2.77	-5.46	-0.45
Electrostatic energy [kcal/mol]	-23249.38	-22311.24	**-23798.96**	-22779.24
Total charge	-19	-20	-20	-21
APBS Solvation energy [kcal/mol]	-9866.09	-9690.88	**-10326.38**	-10230.40
MEP interaction energy (electrostatic interaction)	9.4380	-13.4179	**-18.4713**	3.0079

[1] Ligand-receptor interaction was evaluated by modeling biomolecular solvation through solving the Poisson-Boltzmann equation and related equations, to calculate solvation energies and electrostatic properties of the complex. The APBS (Adaptive Poisson-Boltzmann Solver) software, developed by Nathan Baker in collaboration with J. Andrew McCammon and Michael Holst, was used as plug-in implementation to Vega ZZ package. APBS calculates binding free energies (*APBS Binding energy)* by dividing the binding process up into solvation (*APBS Solvation energy)* and Coulombic components. Intermolecular Coulombic contributions to the solvation energy are assessed using either *APBS Electrostatic Interaction energies* or *Molecular electrostatic potential (MEP) interaction energy.*
